# Connected Health

**DOI:** 10.3389/fdgth.2019.00001

**Published:** 2019-10-16

**Authors:** Constantinos S. Pattichis, Andreas S. Panayides

**Affiliations:** Department of Computer Science, University of Cyprus, Aglandjia, Cyprus

**Keywords:** mHealth, eHealth, uHealth, telemedicine, telehealth

## What is Connected Health?

According to the Merriam-Webster dictionary^1^, the definition of the word “connected” (adjective) is (i) joined or linked together; (ii) having the parts or elements logically linked together. Similarly, according to the same source, the definition of the word health (noun) is (i) the condition of being sound in body, mind, or spirit; (ii) the general condition of the body, (iia) a condition in which someone or something is thriving or doing well, (iib) general condition or state.

Thus, if we join the two corresponding definitions, it can be inferred that connected health is (i) joining or linking together the condition of being sound in body, mind, or spirit; (ii) having the parts or elements logically linked together for the general condition of the body (or state). These two definitions enrich our knowledge and help us to have a better understanding of the term connected health.

Although there is no standard or accepted definition of connected health, several attempts have been made to define it as briefly presented herein. According to references (1, 2), connected health is an umbrella term arrived at to lessen the confusion over the definitions of telemedicine, telehealth, and mHealth. A very similar description is also given for the Connected Health—Mobile Health and Telehealth program of the National Institute of Biomedical Imaging and Bioengineering.^2^ According to the website of the program, it supports the development of enabling technologies that emphasize the integration of wireless technologies with human and biological interfaces. The program includes the development of software and hardware for telehealth and mobile health studies.

The most complete and extensive definition of connected health was given by Caulfield and Donnelly (3). They stated that “Connected Health encompasses terms such as wireless, digital, electronic, mobile, and tele-health and refers to a conceptual model for health management where devices, services or interventions are designed around the patient's needs, and health related data is shared, in such a way that the patient can receive care in the most proactive and efficient manner possible. All stakeholders in the process are ‘connected' by means of timely sharing and presentation of accurate and pertinent information regarding patient status through smarter use of data, devices, communication platforms and people.” This definition will be adopted for the needs of the connected health specialty section.

## Why is Research in Connected Health so Important?

Ongoing rapid advances in information and communication technologies foretell an emerging paradigm in the delivery of advanced healthcare services under the connected health paradigm. Connected Health is a dynamic and fluid area of digital health and largely encompasses a sociotechnical model for healthcare delivery and management targeted to the development efficient and effective interventions for the benefit of the citizen. In this context, connected health enables the support of P's medicine^2^ (4, 5): (i) *Predictive:* digital technologies that automatically capture information to proactively predict, of diagnose, manage, and/or deliver preventive and therapeutic care for the prevention of disease; (ii) *Pre-emptive:* interconnected and intelligent solutions designed with the result in mind; targeting to identify at-risk individuals before the development of disease symptoms via mobile and/or home-based devices designed to monitor vital signs and activities in real time which communicate with personal health record archives and services, and healthcare professionals; (iii) *Personalized medicine:* enabling the offering of the best possible, most optimal and innovative medical treatment and therapies leading to precision medicine (4, 6); (iv) *Participatory:* enriching models with patient-centric information and experiences. According to Golubnitschaja et al. (4) PPPPPM is considered as the “medicine of the future,” which necessitates a paradigm change for the entire spectrum of medical research and services, improved professional- and citizen-targeted education about the new services and new economic and application models for both disease and health care.

## Why is a Cross-Disciplinary Approach to Research in Connected Health Important?

As it is very clearly defined above, connected health is by its nature a cross-disciplinary area. Although significant progress has been made in recent years, much still has to be done to facilitate the deployment of connected health services massively (see also next section).

## Much has been and Continues to be Published in this area. What Specific Boundaries of the Research Urgently need to be Pushed to Offer the Best Outcomes for the Future?

To the best of our knowledge, the research areas that need to be pushed further in order to trigger the best outcomes for the future in the offering of added value connected health services, efforts should be concentrated in the following 5 areas:

- **Sensing and Point of Care Devices:** New sensing and actuation devices (edge devices) and point of care devices including wearables are expected to reduce the frequency of hospital visits, travel expenses, and lost work time. Consider for example the success of glucose meters, which has motivated more people to opt for self-testing and monitor their health status.
- **Communication Protocols and Technologies:** Emerging 5G mobile networks will enable the offering of low-latency, high-reliability, and high-throughput connected health applications. Moreover, connected health applications will be supported by streaming video and immersive media.
- **Architectural Design, Applications, and Interoperability:** Rapid advances in ICT hardware and software portend the development of new architectural designs for connected health applications exploiting the concepts of edge devices, edge gateway, edge and fog computing, and cloud computing service models (software as a service, platform as a service, infrastructure as a service). Fog and edge computing-based applications are network and system architectures that attempt to collect, store, analyze, and process data originating from edge devices more efficiently than the server-side infrastructure, pushing intelligence and processing capabilities down closer to where the data originates at the network edge. Furthermore, new interoperability standards, like the FHIR®–Fast Healthcare Interoperability Resources^3^—should be followed to enable the development of integrated eHealth connected health solutions. FHIR is a cost-effective solution based on the latest web standards, suitable for use in a wide variety of contexts, like mobile apps, cloud communications, and electronic health record-based data sharing.
- **Security and Privacy:** Connected health applications rely on internet connectivity, making them vulnerable and appealing for malicious interventions. Moreover, users' privacy has to be secured, demanding timely investigation and the application of emerging secured and privacy guaranteed protocols, methods, and procedures.
- **Business Added Value:** The wider deployment and penetration of connected health systems and services will significantly affect how medical practice, services and care are provided, making necessary the introduction of new business models capitalizing on the optimal use and exploitation of connected health services.
- **Data Analytics:** Machine learning and deep learning to support the building of smarter health systems that continually analyze information from multiple devices and multiple sources to derive insights and recommendations for the individual's health profiles, as well as AI analytics to monitor mobile device data and use rules and logic to compare against targets, track progress, and send alerts (3).

## What is the Most Important Contribution Research in this area is Going to Make to Other Fields or Disciplines?

It is expected that the massive deployment of personalized connected health systems and services will have a significant impact in our lives as citizens, or patients or consumers. First of all, hopefully, it will help governments to reduce health care costs and improve quality of life and increase the convenience of people. The modus operandi has to be shifted from disease care to health care via the exploitation of the connected health paradigm^2^.

## What do you say When Someone Asks you Why you Dedicate Yourself to This Research?

Connected health is a fast moving cross-disciplinary area, and we dedicate ourselves in working in this emerging research area because we strongly believe and support that advances in the field of connected health systems, technologies, and services will further enable, drive, and accelerate the development, translation, and application of advanced healthcare delivery systems into everyday life and clinical practice toward improved disease management and treatment, enabling precision and preventive healthcare services at reduced cost. Our motive is “*Connected health informatics, systems, technologies, and services for fighting against disease and obtaining better healthcare and well-being.”*

## Author Contributions

All authors listed have made a substantial, direct and intellectual contribution to the work, and approved it for publication.

### Conflict of Interest

The authors declare that the research was conducted in the absence of any commercial or financial relationships that could be construed as a potential conflict of interest.

## Footnotes

^1^ https://www.merriam-webster.com/dictionary/

^2^ https://www.nibib.nih.gov/research-funding/connected-health

^3^ http://hl7.org/fhir/
